# Effects of Ante-Mortem Vitamin D_3_ Supplementation on Meat Quality in Yanbian Yellow Bulls

**DOI:** 10.3390/ani16050818

**Published:** 2026-03-05

**Authors:** Binru Li, Beibei Hao, Hongyan Xu, Xinxin Zhang, Zewen Wu, Bingbing Wang, Yang Yi, Mengxia Sun, Yanzhu Yang, Guangjun Xia

**Affiliations:** 1 Agriculture College, Yanbian University, Yanji 133002, China; 13351727702@163.com (B.L.); haobeibei2021@126.com (B.H.);; 2 Engineering Research Center of North-East Cold Region Beef Cattle Science & Technology Innovation, Yanji 133002, China; 3 Longjing City Animal Disease Prevention and Control Center, Longjing 133400, China

**Keywords:** Yanbian yellow bulls, vitamin D_3_, dose-dependent effects, meat quality

## Abstract

This preliminary study investigated the effects of ante-mortem Vitamin D_3_ (VD_3_) supplementation on blood parameters and meat quality in Yanbian yellow bulls. Twenty healthy Yanbian yellow bulls (intact male Yanbian yellow bulls, 30 ± 1 months of age; initial body weight 534 ± 15 kg) were allocated into five groups: a control (basal diet) and four treatment groups with varying VD_3_ regimens (3 × 10^6^ or 6 × 10^6^ IU/d for 7 days, with or without a 7-day withdrawal). Results demonstrated that, compared to the control group, VD_3_ supplementation elevated serum calcium (*p* < 0.01) and phosphorus levels (*p* < 0.05) while enhancing antioxidant capacity (*p* < 0.05) and immunoglobulin production (*p* < 0.01). Muscle calcium deposition increased (*p* < 0.01), accompanied by higher meat lightness (CIE L*) values (*p* < 0.01) and reduced drip loss rate (*p* < 0.01). Shear force decreased across cuts (*p* < 0.01), which was linked to calpain-mediated protein degradation and sarcomere elongation (*p* < 0.01). Under the conditions of this study, the protocol involving 3.0 × 10^6^ IU/d VD_3_ supplementation for 7 days followed by a 7-day withdrawal yielded the most favorable outcomes. These findings suggest a potential strategy to improve beef quality by regulating calcium homeostasis and enhancing muscle proteolysis, warranting further validation in larger populations.

## Simple Summary

Consumer demand for high-quality beef is increasing. This preliminary study explored a potential strategy to improve the quality of beef from Yanbian yellow bulls by supplementing their diet with vitamin D_3_ before harvest. We observed that giving bulls a specific amount of vitamin D_3_ for a week, followed by a week without supplementation before harvest, was associated with improved meat quality. The beef became more tender and exhibited increased lightness; this effect appears to be mediated by increasing calcium levels in the muscle, which in turn promotes improvements in muscle structure, resulting in more tender meat. Our findings offer preliminary evidence that may assist in developing strategies for producing higher-quality beef.

## 1. Introduction

The escalating consumer demand for premium beef, driven by rising living standards, has necessitated the focus on multifaceted quality benchmarks, including tenderness, color, juiciness, intramuscular fat content, and safety [1]. Beef quality is governed by an intricate multifactorial nexus of production determinants—spanning genetic predisposition, nutritional regimens, and ante-mortem interventions [2,3]—with current research highlighting two key elements: oxidative stability and proteolysis. Current strategies primarily focus on visual and compositional improvements via marbling optimization [4] and vitamin E-mediated lipid stabilization [5,6,7]. However, these approaches often overlook structural tenderization. Notably, ante-mortem VD_3_ supplementation has emerged as a validated intervention that bridges this gap by elevating intramuscular Ca^2+^ concentrations to activate β-calpain, thereby significantly reducing shear force in bovine models [8,9].

Vitamin D, a lipophilic secosteroid, is obtained through dietary intake or synthesized in the skin upon exposure to ultraviolet B (UVB) radiation. Its activation involves two hydroxylation steps: first, hepatic cytochrome P450 2R1 (CYP2R1) converts it into 25-hydroxyVD_3_ (25-OH-D_3_) [10]; then, renal CYP27B1 catalyzes the formation of the biologically active 1,25-dihydroxyVD_3_ (1,25-(OH)_2_-D_3_) [11]. This active metabolite exerts genomic effects by binding to the vitamin D receptor (VDR), which heterodimerizes with the retinoid X receptor (RXR) and recognizes vitamin D response elements (VDREs) in the promoters of target genes [12,13]. Through this pathway, the VDR-RXR complex regulates genes essential for calcium phosphate homeostasis (e.g., TRPV6, CALB1) and immune modulation (e.g., IL10, DEFB4), underscoring vitamin D’s pivotal roles in mineral metabolism and immunoregulation [14].

The bioactive metabolite 1,25-(OH)_2_-D_3_ exerts dual functions in immunomodulation and mineral homeostasis. In the immune system, it enhances B cell differentiation through IL-10 upregulation, resulting in a 2.3-fold increase compared to controls (*p* < 0.01) and stimulating IgG synthesis to bolster humoral immunity [15]. Concurrently, VD_3_ optimizes calcium-phosphorus metabolism by binding to VDR in duodenal enterocytes (upregulating calbindin-D9k expression by 45% at the mRNA level) and by modulating TRPV5 channels in the kidney, thereby reducing urinary Ca^2+^ and phosphorus excretion by 22–35% [16]. These mechanisms associated with measurable improvements in meat quality: for instance, Swanek et al. [17] demonstrated that supplementing with beef steers 5 × 10^6^ IU/d for 7 days increased *longissimus dorsi* Ca^2+^ content and reduced shear force (*p* < 0.01) at 7 days postmortem, with tenderness improvements persisting through 21 days of aging. Karges et al. [18] identified 2.5 × 10^6^ IU/d of VD_3_ as the minimal effective dose for tenderization of the *Gluteus medius* muscle, achieving a significant reduction in shear force (*p* < 0.05), with diminishing returns observed at doses above 5 × 10^6^ IU/d. Muscle-specific responses were also noted, with *Semimembranosus* exhibiting delayed tenderization compared to the more rapid response in Longissimus [19]. Previous studies have shown that high-dose VD_3_ supplementation can improve meat eating quality. Optimal dosages may be breed-dependent; for instance, Montgomery et al. [20] reported that Holstein cattle achieved maximum tenderization at 7.5 × 10^6^ IU/d. The present study therefore aimed to evaluate the effects of different VD_3_ supplementation regimens on meat quality in Yanbian yellow cattle.

Although research on the nutrition of beef cattle has placed a significant emphasis on VD_3_ in recent years, the exact mechanism by which it improves meat quality remains unclear. Previous studies have shown that the optimal dosage and timing of VD_3_ supplementation may vary among cattle breeds. However, limited information is available regarding the effects of VD_3_ on meat quality in indigenous Chinese breeds such as Yanbian yellow cattle. One of China’s five main native breeds, the Yanbian yellow bulls, has considerable potential due to its resilience, ability to withstand roughage, resistance to disease, adaptability, high harvest rate, and excellent meat quality. Significant inter-individual variations in meat tenderness and related quality parameters persist despite the favorable attributes of Yanbian yellow cattle, resulting in inconsistent product quality. Therefore, this study aimed to evaluate the effects of pre-harvest Vitamin D_3_ supplementation on blood biochemical parameters and meat quality attributes in Yanbian yellow cattle. We hypothesized that Vitamin D_3_ supplementation would regulate calcium homeostasis to activate the calpain system and enhance antioxidant capacity, thereby improving meat tenderness and color stability. The findings of this study are intended to provide scientific evidence regarding the physiological and meat quality responses to Vitamin D_3_ in this specific breed, offering a theoretical basis for future nutritional strategies.

## 2. Materials and Methods

All experimental procedures in this experiment were carried out in accordance with the guidelines established by the ‘Laboratory Animal Management Regulations’ (Ministry of Science and Technology, China, 2017) and approved by the Medical Ethics Committee of Yanbian University Medical College (approval No: 201702).

### 2.1. Animals and Treatment Diets

Twenty healthy Yanbian yellow bulls (intact male Yanbian yellow bulls, 30 ± 1 months of age; initial body weight 534 ± 15 kg) were randomly assigned to five dietary treatments (*n* = 4 bulls per treatment): a control group fed a basal total mixed ration (TMR) providing 8000 IU VD_3_/kg dry matter (DM); the A group receiving the basal TMR plus 6 × 10^6^ IU/d VD_3_ per bull daily for 7 d followed by immediate harvest; the B group receiving the same high dose for 7 d, then basal diet only for an additional 7 d before harvest; the C group receiving 3 × 10^6^ IU/d VD_3_ per bulls daily for 7 d followed by immediate harvest; and the D group receiving the same moderate dose for 7 d, then basal diet only for an additional 7 d before harvest. Bulls were housed individually in well-ventilated tie-stalls (3 m × 3 m) bedded with rubber mats and rice straw litter. Ambient temperature was maintained at 15–22 °C with natural light. TMR was offered twice daily (08:00 and 17:00) and water was available ad libitum via automatic drinkers. VD_3_ (cholecalciferol, ≥98% purity) was purchased from Dalian Kunbang Pharmaceutical Co., Ltd., Dalian, China, weighed individually for each bull, and top-dressed onto the evening ration to ensure complete consumption. The ingredient composition and nutrient levels of the basal diet are presented in Table 1.

### 2.2. Harvesting, Collection of Blood and Meat Samples

At the end of the assigned supplementation or withdrawal period, bulls were fasted for 12 h with free access to water and transported 10 km to a commercial abattoir. They were stunned using a captive bolt pistol and exsanguinated following standard commercial procedures. Carcasses were chilled at 4 °C for 48 h. Immediately before harvest, 10 mL of jugular blood was collected into vacuum tubes without anticoagulant, centrifuged at 3000× *g* for 15 min, and the serum was stored at −80 °C. Subsequently, tissue samples were excised from three anatomically defined muscles on the left side of each carcass: the *longissimus dorsi* (between the 12th and 13th ribs), the *deltoid* (cranial portion adjacent to the scapula spine), and the *semimembranosus* (medial aspect of the round near the femur head). Small aliquots (approx. 5 g) of each muscle were minced and flash-frozen in liquid nitrogen for enzyme activity and protein analysis. Larger muscle blocks (approx. 500 g) were vacuum-packed and stored at −20 °C for meat quality trait analysis.

### 2.3. Analysis of Elemental Content and Immunological and Biochemical Indicators

The elemental, immunological, and biochemical parameters of bulls’ blood and *longissimus dorsi* were analyzed by a commercial laboratory (Beijing Sino-UK Biological Technology Co., Beijing, China) using validated commercial assay kits (Nanjing Jiancheng Bioengineering Institute, Nanjing, China) and a semi-automatic biochemical analyzer (model A6, Beijing Shiningsun Technology Inc., Beijing, China). Elemental contents of meat were measured by the same laboratory. Calcium, phosphorus, magnesium, and sodium concentrations were determined by o-cresolphthalein complexone colorimetry (600 nm), ammonium molybdate dual-wavelength colorimetry (340/660 nm), Calmagite complex assay (530/546 nm), and β-galactosidase kinetic assay (405 nm), respectively. Serum albumin was quantified by the bromocresol green method (630 nm); HDL-C and LDL-C by the clearance method (546 nm); triglycerides by the GPO-PAP method (500 nm); and glucose by the GOD-POD method (505 nm). Alanine aminotransferase (ALT) and aspartate aminotransferase (AST) were assayed by continuous monitoring of NADH depletion (340 nm). Glutathione peroxidase (GSH-Px) activity was determined based on the rate of H_2_O_2_-dependent GSH oxidation. Oxidative stress indicators—including total antioxidant capacity (T-AOC), catalase (CAT), superoxide dismutase (SOD), and malondialdehyde (MDA)—were measured using Fe^3+^ reduction (632 nm), H_2_O_2_ decomposition (405 nm), o-cyclobenzotriol autoxidation inhibition (405 nm), and thiobarbituric acid condensation (532 nm), respectively. MDA values were divided by 5 to correct for amplification multiplicity. Immunoglobulins IgG, IgA, and IgM were quantified by an antigen–antibody complex turbidimetric assay.

### 2.4. Analysis of Meat Quality Characteristics

Muscle pH was measured using a pH-STAR direct meter (Matthäus, Eckelsheim, Germany). Meat color was assessed using an OPTO-LAB colorimeter (Matthäus, Germany) for CIE L* (lightness), a* (redness), and b* (yellowness) values after 30 min of blooming at 4 °C. Drip loss was determined by suspending trimmed samples (3 cm × 2 cm × 1 cm, $W_{1}$) in bags at 0–4 °C for 24 h and re-weighing ($W_{2}$); it was calculated as $[(W_{1}\;-\;W_{2})/W_{1}]\;\times \;100$. Cooking loss was evaluated using 2 cm × 2 cm × 2 cm samples ($W_{3}$) heated in a water bath (HH.S11-2, Shanghai Boxun Medical Biological Instrument Corp., Shanghai, China) to an internal temperature of 75–80 °C, cooled, and re-weighed ($W_{4}$); it was calculated as $[(W_{3}\;-\;W_{4})/W_{3}]\;\times \;100$. Meat tenderness was measured with a C-LM3B meter (Tenovo, Beijing, China). Conventional chemical compositions, including moisture, protein, fat, and ash, were determined following National Food Safety Standards GB 5009.3-2016 [21], GB 5009.5-2016 [22], GB 5009.6-2016 [23], and GB 5009.4-2016 [24], respectively.

### 2.5. Measurement of the Electronic Nose

For electronic nose analysis, 5 g of the sample to be measured was accurately weighed into a 20 mL headspace bottle and sealed with a screw cap. The assay temperature was 25 °C, the cleaning time was 90 s, the assay time was 90 s, and the test was repeated five times in parallel. The flavor profiles of different parts of the meat were analyzed using a portable electronic nose system (PEN-3; Airsense Co., Ltd., Schwerin, Germany), and details of the substances sensitive to the E-nose sensor are listed in Table 2. The results were analyzed using Winmuster software (version 1.6.2, copyright Airsense Analytics GmbH, Schwerin, Germany).

### 2.6. Measurement of the Electronic Tongue

The method was based on that of Hongshu Li [25] with slight modification. For this, 2 g of the meat sample to be measured was mixed with 10 mL of distilled water in a pulper for 1 min, followed by centrifugation at 25 °C and 5000× *g* for 10 min, and filtration with filter paper to remove solids, and the filtrate was placed in a 50 mI sample cup and analyzed using an electronic tongue (SA-402B, Insent Co., Seattle, WA, USA). The sensor array consisted of six taste sensors, namely AEE (fresh), CTO (salty), COO (bitter), CAO (sour), AE1 (astringent), and GL1 (sweet), and two reference electrodes. For electronic tongue analysis, the sensor array was first cleaned in cleaning solution for 90 s, then immersed in reference solution for 120 s. This cleaning step was repeated twice. After cleaning, the sensors were equilibrated in the zero position for 30 s before being transferred to the sample cup for a 30 s measurement period. After measurement, the sensors were cleaned twice in reference solution for 3 s each time, followed by an aftertaste measurement in fresh reference solution for 30 s. The cycle was repeated four times.

### 2.7. Determination of Muscle Sarcomere Length

Approximately 2 g of *longissimus dorsi* was weighed into a 50 mL centrifuge tube. Then, 18 mL of 0.25 mol/L sucrose solution, pre-cooled to 4 °C, was added. The mixture was homogenized using a homogenizer (PDS00, GREENPRIMA, London, UK) at 6000× *g* for 1 min under ice-bath conditions. A small aliquot of the homogenate was used to prepare a wet-mount slide for microscopic observation. Sarcomeres were observed and imaged at 1000× magnification using a microscope (FV3000, Olympus, Tokyo, Japan). Thirty images were randomly captured and saved for each sample. The sarcomere length was measured from these images using ImageJ software (v. 1.54r, National Institutes of Health, Bethesda, MD, USA), and the results were averaged.

### 2.8. Calpain Activity Quantification

Calpain activity in the longissimus dorsi muscle was quantified using a commercial assay kit (P0375S, Biyuntian, Shanghai, China) according to the manufacturer’s instructions. Briefly, muscle samples were homogenized in PBS buffer, and the homogenate was centrifuged at 10,000× *g* for 10 min at 4 °C. The supernatant was collected and incubated with HRP-labeled detection antibody in microplate strips for 60 min at 37 °C. After washing, substrate solution was added and incubated for 15 min at 37 °C in the dark. The reaction was terminated by adding stop solution, and absorbance was measured at 450 nm within 15 min. Calpain concentration was calculated using a standard curve.

### 2.9. Determination of Myofibril Fragmentation Index

Approximately 1 g of longissimus dorsi was homogenized twice with 20 mL of ice-cold buffer (0.1 mol/L KCl, 0.02 mol/L K_3_PO_4_, 0.001 mol/L MgCl_2_, 0.001 mol/L EDTA, pH 7.1) for 15 s each, with a 10 s interval. The homogenate was centrifuged at 1000× *g* for 15 min, and the pellet was washed by resuspension and centrifugation four times. The final pellet was resuspended, and its protein concentration was determined using the biuret method and diluted to 0.5 mg/mL. Absorbance was measured at 540 nm using a Spark^®^ 10M multimode microplate reader (Tecan Trading Co., Ltd., Shanghai, China). The MFI value was calculated as the absorbance multiplied by 200.

### 2.10. Statistics and Analyses

Data were initially collated using Excel 2010 and then analyzed with SPSS 27.0 software. Due to the small sample size (*n* = 4 per group), formal normality tests were not performed, as their statistical power is insufficient to reliably detect deviations from normality. However, given that the sample sizes were equal across all groups and that analysis of variance (ANOVA) is generally robust to minor violations of the normality assumption, one-way ANOVA was employed to compare differences among treatment groups. When significant differences were detected, Duncan’s multiple range test was used for post hoc comparisons. Homogeneity of variances was assessed using Levene’s test. Results are expressed as mean ± standard deviation (SD). A probability (*p*) value of less than 0.05 was considered statistically significant, and *p* < 0.01 was considered highly significant.

## 3. Results

### 3.1. Effects of VD_3_ on Serum Mineral Contents, Biochemical Parameters, Antioxidant Markers, and Immune Status in Yanbian Yellow Bulls

VD_3_ administration significantly influenced blood mineral profiles. As shown in Table 3, serum calcium concentrations were highest in Groups A and B (*p* < 0.05), followed by Group D, with Group C exhibiting higher levels than the Control group (*p* < 0.05). No significant differences were observed in serum phosphorus, magnesium, or sodium levels among the groups (*p* > 0.05). Regarding antioxidant markers, catalase (CAT) activity was higher in Group A compared to the control (*p* < 0.01). Glutathione peroxidase (GSH-Px) activity increased in Groups A and B (*p* < 0.01), with Group A showing higher activity than Group B. Furthermore, Group A exhibited higher superoxide dismutase (SOD) activity and total antioxidant capacity (T-AOC) (*p* < 0.01). For immune markers, IgA content was higher (*p* < 0.01) in Group A compared to the control. Additionally, IgG and IgM levels were elevated (*p* < 0.05) in Groups A and B, respectively.

### 3.2. Effects of Vitamin D_3_ on Ca, P, Mg, Na Content, and Antioxidant Capacity on Longissimus Dorsi in Yanbian Yellow Bulls

In terms of muscle quality (Table 4), calcium deposition was generally elevated (*p* < 0.01) in the test groups. In particular, calcium levels were higher in group B than in groups C and D. Additionally, sodium levels were increased in group A (*p* < 0.05). Muscle antioxidant capacity was also elevated Compared to the control group, CAT activity was higher in group A, and SOD activity was also higher in group D (*p* < 0.05), while GSH-Px activity was higher in both groups A and D (*p* < 0.05).

### 3.3. Effect of VD_3_ on the Physical Properties and Conventional Chemical Composition of Yanbian Yellow Beef

**Figure 1:**
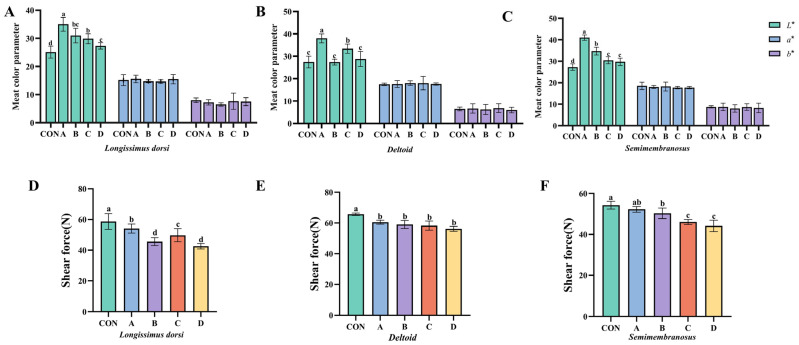
Effects of VD_3_ on meat quality of Yanbian yellow bulls. (**A**–**C**) Meat color parameters (L*, a*, b*) and (**D**–**F**) shear force in the *longissimus dorsi*, *deltoid*, and *semimembranosus* muscles. Data are expressed as mean ± SD. Different letters indicate significant differences (*p* < 0.05).

The effects of Vitamin D_3_ supplementation on meat quality are presented in Table 5 and Figure 1. There were no significant differences in pH values across different muscle parts among the groups (*p* > 0.05).

Meat color analysis (Figure 1A–C) revealed that Vitamin D_3_ supplementation increased lightness (CIE L*) in all muscle parts compared to the control (*p* < 0.05). This effect was most pronounced in the *longissimus dorsi* and *semimembranosus*, where Groups A, B, and C showed significant increases (*p* < 0.01). No significant differences were observed in redness (CIE a*) or yellowness (CIE b*) across muscle parts (*p* > 0.05).

Regarding water-holding capacity, the drip loss rate in the *longissimus dorsi* was lower in all supplemented groups (*p* < 0.01). In the *deltoid* and *semimembranosus*, Groups A, B, and D showed reduced drip loss (*p* < 0.05). Cooking loss rates did not differ significantly among groups (*p* > 0.05).

Shear force analysis (Figure 1D–F) demonstrated a tenderizing effect. In the *longissimus dorsi*, all supplemented groups showed reduced shear force (*p* < 0.05) after 48 h of aging. Group D exhibited the greatest improvement in tenderness across all three muscle cuts tested.

Table 6 presents the conventional chemical composition. No significant differences (*p* > 0.05) were observed among groups for moisture, protein, fat, or ash content in any muscle cut, indicating that Vitamin D_3_ did not alter the basic nutritional composition of the meat.

Overall, these findings confirm that ante-mortem VD_3_ supplementation systematically enhances the tenderness of all muscle parts in Yanbian yellow bulls, with Group D exhibiting the most remarkable improvements. The reduction in shear force across the *longissimus dorsi*, *deltoid*, and *semimembranosus* highlights the efficacy of VD_3_ in improving meat texture. Additionally, supplementation significantly enhances meat brightness, reduces drip loss, and contributes to overall improvements in meat quality, with Groups A and D showing the most promising results for practical application.

### 3.4. Effect of VD_3_ on the Sarcomere Length and the Conventional Chemical Composition in Yanbian Yellow Beef Muscle

**Figure 2:**
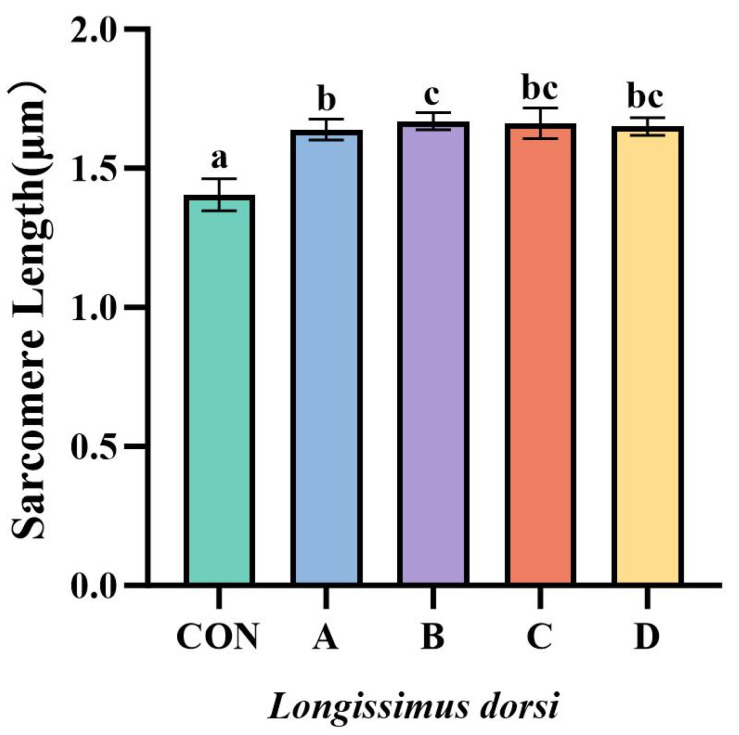
Effect of VD_3_ on sarcomere length in *longissimus dorsi* muscle. Effect of vitamin D_3_ on sarcomere length in longissimus dorsi of Yanbian Yellow bulls. CON, control group fed basal diet. A, group supplemented with 6 × 10^6^ IU/d VD_3_ for 7 days and slaughtered immediately. B, group supplemented with 6 × 10^6^ IU/d VD_3_ for 7 days followed by a 7-day withdrawal before slaughter. C, group supplemented with 3 × 10^6^ IU/d VD_3_ for 7 days and slaughtered immediately. D, group supplemented with 3 × 10^6^ IU/d VD_3_ for 7 days followed by a 7-day withdrawal before slaughter. Data are presented as mean ± SD. Different lowercase letters indicate significant differences (*p* < 0.05). The same letter or no letter indicates no significant difference (*p* > 0.05). Representative microscopic images of sarcomeres from each group are provided in Supplementary Figure S1.

As shown in Figure 2, VD_3_ supplementation significantly increased sarcomere length in all experimental groups compared to the control group (*p* < 0.01; representative images are shown in Supplementary Figure S1). Sarcomere length was also greater in Group B than in Group A (*p* < 0.05), while no significant differences were observed among the remaining groups (*p* > 0.05).

### 3.5. Effects of VD_3_ on Flavor and Odor Substances in Different Parts of Yanbian Yellow Bulls’ Meat

**Figure 3:**
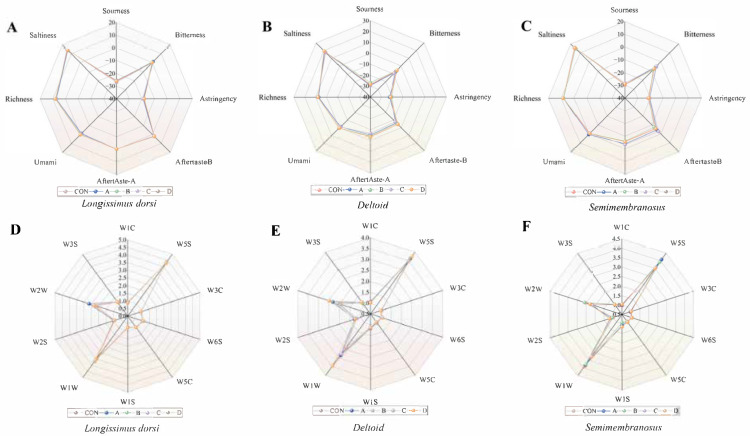
Effect of VD_3_ on flavor and odor substances in various parts of meat. CON, control group fed basal diet. A, group supplemented with 6 × 10^6^ IU/d VD_3_ for 7 days and slaughtered immediately. B, group supplemented with 6 × 10^6^ IU/d VD_3_ for 7 days followed by a 7-day withdrawal before slaughter. C, group supplemented with 3 × 10^6^ IU/d VD_3_ for 7 days and slaughtered immediately. D, group supplemented with 3 × 10^6^ IU/d VD_3_ for 7 days followed by a 7-day withdrawal before slaughter. (**A**) Effect of VD_3_ on flavor substances of longissimus dorsi. (**B**) Effect of VD_3_ on flavor substances of semimembranosus muscle. (**C**) Effect of VD_3_ on flavor substances of deltoid muscle. (**D**) Effect of VD_3_ on odorant substances in longissimus dorsi. (**E**) Effect of VD_3_ on odorant substances in semimembranosus muscle. (**F**) Effect of VD_3_ on odorant substances in deltoid muscle.

Radar plots illustrating the flavor attributes are shown in Figure 3A–C. In the *longissimus dorsi*, basic flavor dimensions such as sourness, bitterness, and astringency showed minimal variation among treatment groups. However, for the astringency index, Groups B and D were higher than Group C (*p* < 0.05). For the freshness dimension, Groups B and D were higher than Groups A and C (*p* < 0.05).

In the *semimembranosus* muscle, the umami sensor response was higher in Group A than in the control and other experimental groups (*p* < 0.05), while no significant differences were observed for the other taste-related sensors (*p* > 0.05).

The *deltoid* muscle presented a more complex profile: for sourness, Group B was higher than the control, Group A, and Group C (*p* < 0.05); for freshness, Groups A and C were lower than the control, Group B, and Group D (*p* < 0.05); and for saltiness, Group D was higher than the control (*p* < 0.05). Other indicators did not differ significantly (*p* > 0.05).

Regarding odor profiles (Figure 3D–F), while numerical variations were observed in specific sensor responses across treatment groups, none of these differences reached statistical significance (*p* > 0.05). This indicates that under the conditions of this study, Vitamin D_3_ supplementation did not significantly alter the overall volatile odor profile of the beef as detected by the electronic nose.

### 3.6. Effect of VD_3_ on the Myogenic Fragmentation Index in Yanbian Yellow Beef

**Figure 4:**
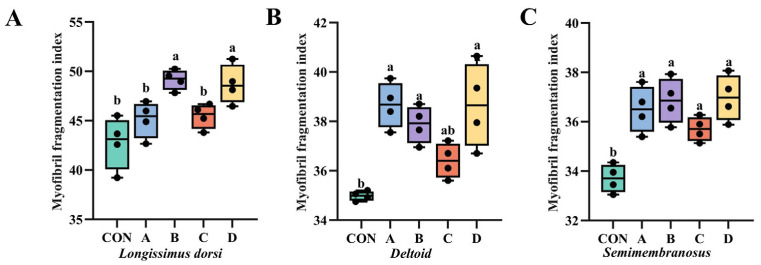
Effect of VD_3_ supplementation on myofibrillar fragmentation index (MFI) in the *longissimus dorsi* muscle of Yanbian yellow bulls. CON, control group fed basal diet. A, group supplemented with 6 × 10^6^ IU/d VD_3_ for 7 days and slaughtered immediately. B, group supplemented with 6 × 10^6^ IU/d VD_3_ for 7 days followed by a 7-day withdrawal before slaughter. C, group supplemented with 3 × 10^6^ IU/d VD_3_ for 7 days and slaughtered immediately. D, group supplemented with 3 × 10^6^ IU/d VD_3_ for 7 days followed by a 7-day withdrawal before slaughter. (**A**) MFI in longissimus dorsi. (**B**) MFI in deltoid muscle. (**C**) MFI in semimembranosus muscle. Different lowercase letters indicate significant differences (*p* < 0.05). The same letter indicates no significant difference.

The effect of VD_3_ on the myofibrillar fragmentation index (MFI) of Yanbian yellow beef is shown in Figure 4. Supplementary feeding of VD_3_ improved the MFI in this beef. Compared to the control group, the MFI in the *longissimus dorsi* was higher in Groups B and D (*p* < 0.05), with no significant differences observed among the remaining groups (*p* > 0.05). Similarly, for *deltoid*, the MFI in Groups A, B, and D was elevated relative to the control (*p* < 0.05). In *semimembranosus*, all groups exhibited a higher MFI than the control group (*p* < 0.05), while no significant differences were found among the groups themselves (*p* > 0.05).

### 3.7. Effect of VD_3_ on the Concentration of Calpain in Yanbian Yellow Bulls

**Figure 5:**
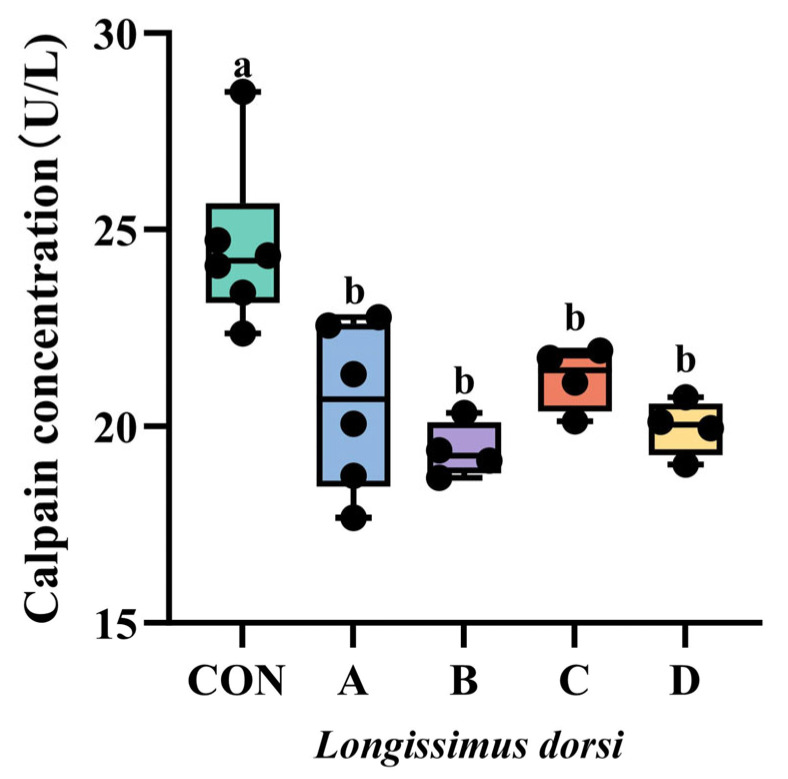
Effect of VD_3_ supplementation on myofibrillar fragmentation index in the *longissimus dorsi* muscle of Yanbian yellow bulls. CON, control group fed basal diet. A, group supplemented with 6 × 10^6^ IU/d VD_3_ for 7 days and slaughtered immediately. B, group supplemented with 6 × 10^6^ IU/d VD_3_ for 7 days followed by a 7-day withdrawal before slaughter. C, group supplemented with 3 × 10^6^ IU/d VD_3_ for 7 days and slaughtered immediately. D, group supplemented with 3 × 10^6^ IU/d VD_3_ for 7 days followed by a 7-day withdrawal before slaughter. Different lowercase letters indicate significant differences (*p* < 0.05). The same letter indicates no significant difference.

The effect of VD_3_ supplementation on *Longissimus dorsi* calpain concentration in Yanbian yellow bulls is shown in Figure 5, from which it can be seen that the calpain concentration in all experimental groups was reduced compared with the control group (*p* < 0.05), but there was no significant difference between the experimental groups (*p* > 0.05).

## 4. Discussion

Ante-mortem VD_3_ supplementation yielded multifaceted improvements in blood chemistry, antioxidant status, immunity, and meat quality of Yanbian Yellow bulls.

Mineral metabolism and serum chemistry parameters were consistent with previous findings in beef cattle [18,20], indicating that VD_3_ elevates serum calcium and phosphorus concentrations. This reflects reduced renal excretion, enhanced intestinal absorption, and increased tubular reabsorption mediated by vitamin D receptor signaling [26,27]. Calcium levels remained consistently higher in the low-dose supplementation with 7-day withdrawal group (Group D) than in the low-dose supplementation without withdrawal group (Group C), with the greatest increases observed in the high-dose groups (Groups A and B), indicating a dose–response relationship and supporting the conclusion that “maximum mineral retention requires gastrointestinal conversion to 1,25-(OH)_2_-D_3_” [9,28]. Serum magnesium levels showed a slight but non-statistically significant decrease, contrary to reports of magnesium depletion induced by ultra-high-dose VD_3_ [29]. Other routine biochemical parameters remained unchanged, indicating no adverse metabolic effects.

VD_3_ significantly enhanced antioxidant capacity. Bulls supplemented with VD_3_ for 7 consecutive days (Groups A and B) exhibited higher glutathione peroxidase and catalase activities, with the high-dose continuous supplementation group (Group A) showing the strongest superoxide dismutase activity and total antioxidant capacity. No significant differences were detected in the response values of any electronic nose sensors among the treatment groups (*p* > 0.05), indicating that VD_3_ supplementation did not substantially alter the volatile aroma profile of Yanbian yellow bulls. VD_3_ reinforced the hepatic antioxidant system by downregulating proinflammatory cytokines and oxidative stress markers [13]. Levels of immunoglobulins IgA, IgG, and IgM also increased concurrently, consistent with 1,25-(OH)_2_-D_3_-induced B-cell differentiation and IL-10-mediated antibody class switching [15]. These findings confirm that VD_3_ concurrently supports both humoral and cellular immune functions.

The physical properties, sarcomere length, and conventional chemical composition of beef are core dimensions for evaluating its meat quality. The regulatory effects of VD_3_ supplementation on these three indicators are closely associated with Ca^2+^-mediated metabolic mechanisms [30,31]. The color of meat is primarily regulated by myoglobin content and its redox state. pH is closely related to ante-mortem meat tenderness, flavor, and water-holding capacity. Cooking loss and drip loss are key indicators reflecting water retention. Tenderness is influenced by multiple factors acting synergistically, including the characteristics of intramuscular connective tissue, intramuscular fat content, and the degree of myofibrillar protein hydrolysis [32,33]. Previous studies have demonstrated that ante-mortem supplementation with VD_3_ significantly reduces beef shear force values, improves water-holding capacity, and enhances meat color brightness, without significantly affecting pH levels [34]. The results of this experiment align closely with this conclusion. Specifically, supplemental VD_3_ did not significantly alter the pH value or cooking loss rate of Yanbian yellow beef. However, it significantly increased the CIE L* value of meat from all cuts, significantly reduced drip loss, and markedly decreased shear force. Notably, experimental groups B and C exhibited a highly significant reduction in shear force for the sirloin cut. Group D demonstrated superior improvements in tenderness for shoulder and leg cuts. The core regulatory mechanism is hypothesized to involve elevated VD_3_ levels enhancing Ca^2+^ concentration in muscle tissue postmortem [20]. This action simultaneously weakens myofibrillar structural integrity and activates the calpain system to promote myofibrillar protein degradation, ultimately achieving meat tenderization. Sarcomere, as the smallest contractile units of striated muscle, exhibit a close correlation between their length and beef tenderness—increased sarcomere length can significantly reduce meat shear force, while sarcomere condition also influences muscle water-holding capacity [35]. In this experiment, the sarcomere length of Yanbian Yellow Cattle in all experimental groups showed a highly significant increase. This result may be attributed to VD_3_ increasing muscle Ca^2+^ concentration, activating the calpain system, and enhancing protease activity [36]. Research by England et al. [37] also confirms that increased sarcomere length promotes post-harvest tenderization in meat. This indicates that sarcomere length not only directly influences tenderness but also indirectly affects the degradation process of myofibrillar skeletal proteins by regulating the contact efficiency between μ-calpain and its substrates [38]. However, the mechanism underlying the interaction between sarcomere length and the calpain system during postmortem rigor mortis remains to be further investigated. At the level of conventional chemical composition, the nutritional value of beef is primarily determined by its moisture, protein, fat, and ash content [39,40]. This study found that ante-mortem supplementation with VD_3_ had no significant effect on the conventional chemical composition of various cuts of Yanbian Yellow cattle meat. Differences in moisture content among cuts primarily stemmed from variations in the looseness of muscle fiber and protein tissue structure. These results indicate that while VD_3_ can improve meat tenderness by increasing Ca^2+^ concentration and activating the calpain system, it does not alter the core nutritional composition of beef, thereby ensuring the stability of its edible value.

Electronic tongues and electronic noses can respectively analyze the sensory characteristics of Yanbian yellow beef from the dimensions of taste and aroma [41]. The umami flavor of the meat primarily originates from water-soluble precursors in muscle tissue, with free amino acids exerting the most critical influence [42]. Their concentrations are closely correlated with flavor intensity. Specifically, glutamic acid and aspartic acid are the primary contributors to umami taste, especially when synergized with nucleotides. Alanine, glycine, serine, and proline are the predominant sweet-tasting amino acids, while branched-chain amino acids such as isoleucine and leucine significantly influence bitterness perception [43]. Electronic tongue analysis revealed that VD_3_ supplementation did not significantly alter the taste profiles of Yanbian yellow beef, as no consistent differences were observed among treatment groups for any of the taste-related sensors (*p* > 0.05). Similarly, electronic nose analysis showed no significant differences in sensor responses among treatment groups for any of the muscles examined (*longissimus dorsi*, *deltoid*, *semimembranosus*) (*p* > 0.05). These results indicate that, under the conditions of this study, VD_3_ supplementation did not substantially affect the volatile aroma profiles of Yanbian yellow beef.

The integrity and degradation level of muscle myofibrils are commonly represented by the myofibrillar fragmentation index (MFI) value. Changes in MFI correlate with the degradation of myofibrillar proteins. MFI values alter with the disruption of myofibrillar structure; higher MFI values indicate better muscle tenderness. Compared to the control group, all VD_3_-supplemented groups exhibited significantly higher myofibrillar fragmentation index (MFI) values in the *semimembranosus* muscle (*p* < 0.05). For the *longissimus dorsi*, higher MFI values were observed in Groups A, B, and D, while the *deltoid* muscle showed significant increases in Groups A, B, and D (*p* < 0.05). These findings are consistent with Gonzalez et al. [44], who reported that calcium ion treatment enhances beef MFI. This effect is likely mediated by elevated intracellular Ca^2+^ activating the calpain system [45], leading to degradation of Z-line skeletal proteins [46].

The calpain system plays a pivotal role in the tenderization process of post-harvest muscle [47]. Within muscle tissue, the calpain system regulates myofibrillar protein degradation, which constitutes the rate-limiting step in this process. Consequently, the calpain system is crucial for meat tenderization [48]. During muscle maturation, m-calpain activity remains unchanged while μ-calpain activity decreases. This may be attributed to the low Ca^2+^ concentration (100 μmol/L) in living cells, which is insufficient to activate m-calpain [49]. The decrease in calpain activity is considered a manifestation of its role in hydrolyzing myofibrillar protein, making μ-calpain the primary enzyme influencing meat tenderness changes. This study indicates that compared to the control group, calpain concentrations were significantly reduced in all experimental groups, though no significant differences existed among the experimental groups. These results are consistent with those of Montgomery et al. [50], who administered vitamin D_3_ at doses of 0, 0.5 × 10^6^, 1 × 10^6^, and 5 × 10^6^ IU/d to beef cattle for 8 days prior to harvest. Their findings showed that μ-calpain activity in the experimental groups was significantly lower than in the control group, with no significant differences among the experimental groups, while m-calpain activity was not significantly affected.

It is important to acknowledge the limitations of this study, most notably the relatively small sample size (*n* = 4 per group). This constraint was primarily due to the practical and logistical challenges associated with conducting controlled feeding trials in large ruminants. However, the sample size in the present study is comparable to that used in other published studies investigating similar physiological responses in beef cattle [51]. While this sample size inherently limits statistical power and generalizability, the consistent significant differences observed in key markers (calcium, shear force) suggest robust biological effects. Therefore, the results of this study should be interpreted as a preliminary investigation. Future validation through large-scale studies involving a greater number of animals from diverse genetic backgrounds is recommended to confirm these observations.

## 5. Conclusions

In conclusion, ante-mortem supplementation with Vitamin D_3_ proved to be an effective strategy to improve the meat quality of Yanbian yellow bulls. The treatment significantly elevated blood calcium levels and antioxidant enzyme activities (SOD, GSH-Px, CAT). These physiological changes resulted in decreased shear force (improved tenderness) through the activation of the calpain system, as well as reduced drip loss and increased meat lightness (L*) due to enhanced antioxidant status. Among the tested dosages, the administration of Vitamin D_3_ (Group D, 3 × 10^6^ IU/d with a 7-day withdrawal) showed the most beneficial effects on meat quality attributes without altering the basic chemical composition of the meat. These findings suggest that supranutritional Vitamin D_3_ supplementation can be applied in the finishing phase to enhance the market value of Yanbian yellow bulls.

## Figures and Tables

**Table 1:** Ingredient and nutritional composition of basal diets (%, dry matter basis).

Ingredient Composition	Content, %	Nutrient Levels	Content, %
Corn Soybean meal Wheat bran NaHCO_3_ NaCl Permix ^1^ Total	64.0 19.2 10.0 1.0 0.8 5.0 100	Crude protein Crude fat Crude ash Neutral detergent fibers Acid detergent fibers Calcium Phosphorus Net weight gain ^2^ (MJ/kg)	20.70 4.46 4.07 18.43 12.06 0.74 0.57 4.37

^1^ Provided per kg of concentrate: VD_3_ 80,000 IU, vitamin E 200 mg, vitamin K 10 mg, biotin 2 mg, Fe 2000 mg, Mn 2000 mg, Zn 2000 mg, Cu 400 mg, I 16 mg, Se 100 mg, Co 20 mg, ethoxyquin 500 mg. ^2^ Net energy for gain (NEg) was calculated based on ingredient composition, while all other nutrient levels were measured values.

**Table 2:** PEN-3 electronic nasal (E-nose) sensor-sensitive substances.

Array Number	Sensor	Substances for Sensing
R1	W1C	Aromatic hydrocarbons
R2	W5S	Nitrogen oxides
R3	W3C	Ammonia and other odorous components
R4	W6S	Hydrides
R5	W5C	Aromatic short-chain alkanes
R6	W1S	Alkanes
R7	W1W	Inorganic sulfides
R8	W2S	Alcohols, aldehydes and ketones
R9	W2W	Aromatic compounds, organosulfur compounds
R10	W3S	Long-chain alkanes

**Table 3:** Effects of VD_3_ on serum mineral contents, biochemical parameters, antioxidant markers, and immune status in Yanbian yellow bulls.

Items ^1^	Groups					
	CON ^2^	A ^3^	B ^4^	C ^5^	D ^6^	*p*-Value ^7^
Ca, mg/dL	8.49 ± 0.06 ^d^	10.60 ± 0.09 ^a^	10.78 ± 0.09 ^a^	9.57 ± 0.08 ^c^	9.88 ± 0.09 ^b^	<0.001
P, mg/dL	6.40 ± 0.18	7.37 ± 0.19	7.42 ± 0.41	6.98 ± 0.34	7.11 ± 0.41	0.232
Mg, mg/dL	1.91 ± 0.27	1.65 ± 0.23	1.64 ± 0.22	1.57 ± 0.03	1.32 ± 0.14	0.156
Na, mg/dL	301.83 ± 1.34	304.36 ± 5.10	298.13 ± 6.86	292.70 ± 6.60	294.85 ± 12.46	0.790
ALB, g/L	32.72 ± 0.26	31.65 ± 4.25	27.90 ± 10.44	34.47 ± 5.16	33.43 ± 3.71	0.411
GLB, g/L	32.32 ± 5.12	44.74 ± 5.61	43.76 ± 5.70	35.58 ± 0.44	37.59 ± 6.20	0.605
HDL, mmol/L	1.33 ± 0.17	1.40 ± 0.21	1.09 ± 0.01	1.27 ± 0.36	1.28 ± 0.24	0.801
LDL, mmol/L	0.70 ± 0.01	0.74 ± 0.04	0.72 ± 0.23	0.67 ± 0.02	0.71 ± 0.11	0.341
vLDL, mmol/L	0.42 ± 0.03	0.43 ± 0.05	0.46 ± 0.09	0.47 ± 0.02	0.53 ± 0.06	0.591
TG, mmol/L	0.25 ± 0.02	0.20 ± 0.05	0.25 ± 0.02	0.23 ± 0.05	0.26 ± 0.04	0.315
GLU, mmol/L	3.46 ± 0.77	3.51 ± 0.62	3.88 ± 0.97	3.49 ± 0.44	3.72 ± 0.04	0.864
ALT, U/L	22.83 ± 1.80	21.62 ± 2.53	22.89 ± 2.32	22.70 ± 8.62	24.46 ± 4.00	0.689
AST, U/L	74.50 ± 3.51	75.17 ± 4.71	77.84 ± 3.78	75.37 ± 1.67	77.31 ± 2.95	0.207
CAT, U/mL	40.07 ± 0.86 ^b^	46.77 ± 3.28 ^a^	42.11 ± 1.15 ^a^	39.29 ± 1.56 ^b^	39.77 ± 2.11 ^b^	<0.001
GSH-PX, U/mL	380.98 ± 4.77 ^c^	452.66 ± 10.19 ^a^	421.30 ± 2.34 ^b^	396.22 ± 2.90 ^c^	395.34 ± 0.86 ^c^	<0.001
SOD, U/mL	66.95 ± 2.65 ^b^	78.33 ± 7.22 ^a^	75.27 ± 3.38 ^b^	66.29 ± 3.15 ^b^	68.65 ± 2.66 ^b^	<0.001
T-AOC, U/mL	11.92 ± 0.34 ^b^	14.10 ± 1.12 ^a^	12.31 ± 0.37 ^b^	12.30 ± 0.39 ^b^	11.85 ± 0.15 ^b^	<0.001
IgA, g/L	1.55 ± 0.02 ^b^	1.93 ± 0.04 ^a^	1.73 ± 0.03 ^ab^	1.58 ± 0.06 ^b^	1.50 ± 0.04 ^b^	<0.001
IgG, g/L	9.72 ± 0.13 ^b^	12.84 ± 1.18 ^a^	11.78 ± 1.67 ^a^	9.78 ± 0.18 ^b^	9.72 ± 0.19 ^b^	<0.001
IgM, g/L	1.17 ± 0.02 ^b^	1.33 ± 0.11 ^a^	1.32 ± 0.17 ^a^	1.19 ± 0.06 ^b^	1.17 ± 0.06 ^b^	<0.001

^1^ Ca, Calcium; P, Phosphorus; Mg, Magnesium; Na, Sodium; ALB, Albumin; GLB, Globulin; HDL, High-Density Lipoprotein; LDL, Low-Density Lipoprotein; vLDL, Very Low-Density Lipoprotein; TG, Triglycerides; GLU, Glucose; ALT, Alanine Aminotransferase; AST, Aspartate Aminotransferase; CAT, Catalase; GSH-PX, Glutathione Peroxidase; SOD, Superoxide Dismutase; T-AOC, Total Antioxidant Capacity; IgA, Immunoglobulin A; IgG, Immunoglobulin G; IgM, Immunoglobulin M. ^2^ CON, bulls fed the control diet. ^3^ A, bulls fed 6 × 10^6^ IU/d vitamin D_3_ for 7 d and harvested immediately. ^4^ B, bulls fed 6 × 10^6^ IU/d vitamin D_3_ for 7 d, followed by a 7 d withdrawal period before harvest. ^5^ C, bulls fed 3 × 10^6^ IU/d vitamin D_3_ for 7 d and harvested immediately. ^6^ D, bulls fed 3 × 10^6^ IU/d vitamin D_3_ for 7 d, followed by a 7 d withdrawal period before harvest. ^7^ *p* value, *p*-value of control group and experimental group. Values are presented as mean ± SD. Means in the same row with different lowercase superscripts (a–d) differ significantly (*p* < 0.05).

**Table 4:** Effects of vitamin D_3_ on mineral content and antioxidant indexes of *longissimus dorsi* in Yanbian yellow bulls.

Items ^1^	Groups					
	CON ^2^	A ^3^	B ^4^	C ^5^	D ^6^	*p*-Value ^7^
Ca mg/100 g	8.78 ± 0.20 ^d^	11.40 ± 0.11 ^ab^	11.88 ± 0.23 ^a^	10.50 ± 0.31 ^c^	11.14 ± 0.08 ^b^	<0.001
P mg/100 g	64.62 ± 2.80	76.48 ± 1.58	71.33 ± 7.97	75.89 ± 10.46	71.96 ± 9.64	0.207
Mg mg/100 g	19.89 ± 1.97	20.81 ± 0.07	21.10 ± 1.21	22.41 ± 0.29	20.45 ± 0.34	0.428
Na mg/100 g	3036.91 ± 109.97 ^b^	3293.80 ± 144.47 ^a^	3187.37 ± 42.04 ^ab^	3209.01 ± 28.61 ^ab^	3112.26 ± 140.80 ^ab^	<0.001
T-AOC, U/mL	5.73 ± 0.33 ^b^	6.33 ± 0.36 ^a^	6.59 ± 0.22 ^a^	5.70 ± 0.41 ^b^	6.56 ± 0.24 ^a^	<0.001
CAT, U/mL	26.21 ± 4.91 ^b^	36.88 ± 2.01 ^a^	32.52 ± 6.59 ^ab^	30.96 ± 6.67 ^ab^	31.50 ± 2.63 ^ab^	0.01
SOD, U/mL	64.03 ± 1.82 ^b^	67.51 ± 1.27 ^ab^	72.84 ± 3.61 ^a^	64.59 ± 4.11 ^b^	70.65 ± 3.51 ^a^	<0.001
GSH-PX, U/mL	191.82 ± 2.29 ^b^	231.64 ± 11.16 ^a^	203.10 ± 5.93 ^b^	204.16 ± 7.31 ^b^	242.89 ± 9.78 ^a^	<0.001
MDA, nmol/mL	6.87 ± 0.56	6.19 ± 0.34	6.23 ± 0.32	6.71 ± 0.66	6.29 ± 0.35	0.129

^1^ Ca, Calcium; P, Phosphorus; Mg, Magnesium; Na, Sodium; T-AOC, Total Antioxidant Capacity; CAT, Catalase; SOD, Superoxide Dismutase; GSH-PX, Glutathione Peroxidase; MDA, Malondialdehyde. ^2^ CON, bulls fed the control diet. ^3^ A, bulls fed 6 × 10^6^ IU/d vitamin D_3_ for 7 d and harvested immediately. ^4^ B, bulls fed 6 × 10^6^ IU/d vitamin D_3_ for 7 d, followed by a 7 d withdrawal period before harvest. ^5^ C, bulls fed 3 × 10^6^ IU/d vitamin D_3_ for 7 d and harvested immediately. ^6^ D, bulls fed 3 × 10^6^ IU/d vitamin D_3_ for 7 d, followed by a 7 d withdrawal period before harvest. ^7^ *p* value, *p*-value of control group and experimental group. Values are presented as mean ± SD. Means in the same row with different lowercase superscripts (a–d) differ significantly (*p* < 0.05).

**Table 5:** Effect of VD_3_ on the meat trait of Yanbian yellow beef.

Items ^1^	Departments	Groups					
		CON ^2^	A ^3^	B ^4^	C ^5^	D ^6^	*p*-Value ^7^
pH	*Longissimus* *dorsi*	5.41 ± 0.01	5.46 ± 0.08	5.42 ± 0.02	5.40 ± 0.02	5.46 ± 0.01	0.214
	*Deltoid*	5.66 ± 0.02	5.58 ± 0.01	5.56 ± 0.02	5.68 ± 0.10	5.66 ± 0.15	0.272
	*Semimembranosus*	5.52 ± 0.06	5.50 ± 0.17	5.55 ± 0.12	5.53 ± 0.05	5.51 ± 0.06	0.458
Drip Loss (%)	*Longissimus* *dorsi*	9.69 ± 0.49 ^a^	7.12 ± 0.49 ^b^	7.11 ± 0.62 ^b^	7.66 ± 0.86 ^b^	7.28 ± 0.53 ^b^	<0.01
	*Deltoid*	8.36 ± 0.49 ^a^	7.04 ± 0.85 ^b^	7.09 ± 1.06 ^b^	7.47 ± 0.13 ^a^	6.97 ± 0.30 ^b^	<0.01
	*Semimembranosus*	8.58 ± 0.25 ^a^	7.22 ± 1.21 ^bc^	6.87 ± 0.97 ^bc^	7.71 ± 0.67 ^ab^	6.77 ± 0.04 ^c^	0.007
Cooking Loss (%)	*Longissimus* *dorsi*	28.02 ± 2.32	27.55 ± 1.12	27.02 ± 1.76	28.01 ± 1.13	27.04 ± 0.88	0.461
	*Deltoid*	31.44 ± 5.90	29.83 ± 3.48	29.69 ± 1.42	29.62 ± 1.15	28.59 ± 5.48	0.964
	*Semimembranosus*	30.41 ± 0.87	29.26 ± 2.47	31.38 ± 1.40	31.34 ± 2.91	29.82 ± 0.44	0.740

^1^ pH, pH value; Drip loss, Drip loss percentage; Cooking loss, Cooking loss percentage. ^2^ CON, bulls fed the control diet. ^3^ A, bulls fed 6 × 10^6^ IU/d vitamin D_3_ for 7 d and harvested immediately. ^4^ B, bulls fed 6 × 10^6^ IU/d vitamin D_3_ for 7 d, followed by a 7 d withdrawal period before harvest. ^5^ C, bulls fed 3 × 10^6^ IU/d vitamin D_3_ for 7 d and harvested immediately. ^6^ D, bulls fed 3 × 10^6^ IU/d vitamin D_3_ for 7 d, followed by a 7 d withdrawal period before harvest. ^7^ *p* value, *p*-value of control group and experimental group. Values are presented as mean ± SD. Means in the same row with different lowercase superscripts (a–c) differ significantly (*p* < 0.05).

**Table 6:** Effect of vitamin D_3_ on the conventional chemical composition of Yanbian yellow beef.

Items ^1^	Departments	Groups					
		CON ^2^	A ^3^	B ^4^	C ^5^	D ^6^	*p*-Value ^7^
Moisture, %	*Longissimus* *dorsi*	67.04 ± 1.33	68.90 ± 1.57	68.02 ± 1.15	67.10 ± 1.15	67.69 ± 0.96	0.399
	*Deltoid*	71.18 ± 2.84	72.47 ± 1.28	72.37 ± 0.39	71.91 ± 0.92	72.43 ± 1.26	0.896
	*Semimembranosus*	65.37 ± 0.65	65.88 ± 1.67	66.05 ± 1.32	65.91 ± 1.17	66.30 ± 0.63	0.830
Protein, % of DM	*Longissimus* *dorsi*	54.70 ± 0.63	55.01 ± 1.40	56.24 ± 1.54	55.34 ± 0.82	55.49 ± 1.07	0.930
	*Deltoid*	54.55 ± 1.99	55.35 ± 0.95	54.56 ± 0.49	55.77 ± 0.89	55.52 ± 0.98	0.723
	*Semimembranosus*	54.64 ± 0.56	54.99 ± 0.70	55.16 ± 0.92	55.32 ± 1.11	54.91 ± 0.76	0.658
Fat, % of DM	*Longissimus* *dorsi*	15.68 ± 1.47	15.82 ± 1.29	15.75 ± 1.03	16.03 ± 1.24	15.88 ± 0.74	0.976
	*Deltoid*	9.99 ± 1.23	9.65 ± 0.55	10.34 ± 0.85	9.60 ± 0.89	10.12 ± 1.48	0.794
	*Semimembranosus*	8.56 ± 0.60	8.45 ± 0.36	8.81 ± 0.93	8.72 ± 0.58	8.46 ± 0.81	0.656
Ash, % of DM	*Longissimus* *dorsi*	2.76 ± 0.16	2.71 ± 0.15	2.75 ± 0.11	2.74 ± 0.14	2.70 ± 0.17	0.384
	*Deltoid*	2.67 ± 0.18	2.64 ± 0.29	2.69 ± 0.25	2.53 ± 0.15	2.64 ± 0.11	0.742
	*Semimembranosus*	2.59 ± 0.12	2.70 ± 0.15	2.72 ± 0.17	2.69 ± 0.22	2.71 ± 0.19	0.252

^1^ Moisture, Moisture content; Protein, Crude protein content; Fat, Crude fat content; Ash, Ash content. ^2^ CON, bulls fed the control diet. ^3^ A, bulls fed 6 × 10^6^ IU/d vitamin D_3_ for 7 d and harvested immediately. ^4^ B, bulls fed 6 × 10^6^ IU/d vitamin D_3_ for 7 d, followed by a 7 d withdrawal period before harvest. ^5^ C, bulls fed 3 × 10^6^ IU/d vitamin D_3_ for 7 d and harvested immediately. ^6^ D, bulls fed 3 × 10^6^ IU/d vitamin D_3_ for 7 d, followed by a 7 d withdrawal period before harvest. ^7^ *p* value, *p*-value of control group and experimental group. Values are presented as mean ± SD.

## Data Availability

The original contributions presented in this study are included in the article. Further inquiries can be directed to the corresponding author.

## Supplementary Materials

The following supporting information can be downloaded at: https://www.mdpi.com/article/10.3390/ani16050818/s1, Figure S1: Representative microscopic images of sarcomeres from *longissimus dorsi* of Yanbian Yellow bulls. (A) Control group fed basal diet. (B) Group supplemented with 6 × 10^6^ IU/d VD_3_ for 7 days and slaughtered immediately. (C) Group supplemented with 6 × 10^6^ IU/d VD_3_ for 7 days followed by a 7-day withdrawal before slaughter. (D) Group supplemented with 3 × 10^6^ IU/d VD_3_ for 7 days and slaughtered immediately. (E) Group supplemented with 3 × 10^6^ IU/d VD_3_ for 7 days followed by a 7-day withdrawal before slaughter. Images were captured at 1000× magnification under oil immersion using an Olympus FV3000 microscope. Scale bar = 20 μm. Measured sarcomere lengths (μm) are indicated on the images.
