# Supplementary material for: Effects of Ante-Mortem Vitamin D3 Supplementation on Meat Quality in Yanbian Yellow Bulls
Source: Animals (Basel). 2026 Mar 5;16(5):818. doi: 10.3390/ani16050818 (PMC12984888; doi:10.3390/ani16050818)
Supplement: Supplementary file 1 [file animals-16-00818-s001.zip › animals-4145520-supplementary.pdf]

Supplementary Materials

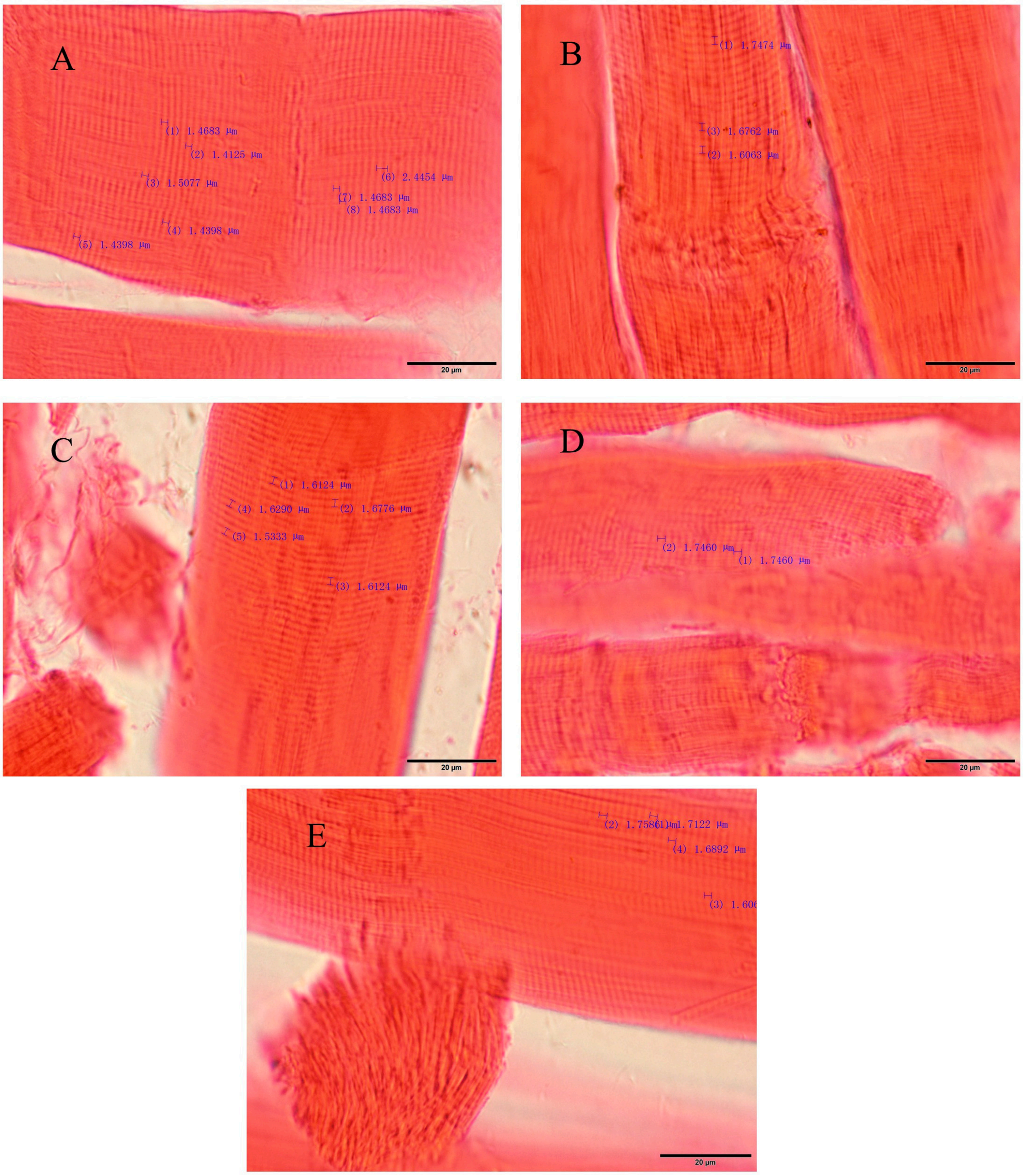

Figure S1. Representative microscopic images of sarcomeres from *longissimus dorsi* of Yanbian Yellow bulls. (A) Control group fed basal diet. (B) Group supplemented with 6×10^6 IU/d VD_3 for 7 days and slaughtered immediately. (C) Group supplemented with 6×10^6 IU/d VD_3 for 7 days followed by a 7-day withdrawal before slaughter. (D) Group supplemented with 3×10^6 IU/d VD_3 for 7 days and slaughtered immediately. (E) Group supplemented with 3×10^6 IU/d VD_3 for 7 days followed by a 7-day withdrawal before slaughter. Images were captured at 1000× magnification under oil immersion using an Olympus FV3000 microscope. Scale bar = 20 µm. Measured sarcomere lengths (µm) are indicated on the images.
